# Supplementary material for: Mycobacterium tuberculosis precursor rRNA as a measure of treatment-shortening activity of drugs and regimens
Source: Nat Commun. 2021 May 18;12:2899. doi: 10.1038/s41467-021-22833-6 (PMC8131613; doi:10.1038/s41467-021-22833-6)
Supplement: Supplementary file 1 — Supplementary Information [file 41467_2021_22833_MOESM1_ESM.pdf]

SUPPLEMENTARY INFORMATION

Mycobacterium tuberculosis precursor rRNA as a measure of treatment-shortening activity of drugs and regimens

Nicholas D Walter,* Sarah E M Born,* Gregory T Robertson,* Matthew Reichlen, Christian Dide-Agossou, Victoria A Ektnitphong, Karen Rossmassler, Michelle E Ramey, Allison A. Bauman, Victor Ozols, Shelby C Bearrows, Gary Schoolnik, Gregory Dolganov, Benjamin Garcia, Emmanuel Musisi, William Worodria, Laurence Huang, J Lucian Davis, Nhung V Nguyen, Hung V Nguyen, Anh TV Nguyen, Ha Phan, Carol Wilusz, Brendan K Podell, N'Dira Sanoussi, Bouke De Jong, Corinne S Merle, Dissou Affolabi, Helen McIlleron, Maria Garcia-Cremades, Ekaterina Maidji, Franceen Eshun-Wilson, Brandon Aguilar-Rodriguez, Dhuvarakesh Karthikeyan, Khisimuzi Mdluli, Cathy Bansbach, Anne J Lenaerts, Rada Savic, Payam Nahid, Joshua J Vásquez, Martin I Voskuil

* These authors contributed equally to this work (NDW, SEMB, GTR)

Contents

Supplementary Methods 2

RNA extraction from *in vitro* samples 2

RNA extraction from murine samples 3

RNA extraction from human samples 3

Nucleic acid radiolabeling 4

Ethics statement and institutional review boards 5

Supplementary Results 6

Supplementary Fig. 1. RS ratio correlates with *Mtb* replication as measured by ITS1/23S ratio 6

Supplementary Fig. 2. Replicate results for ISH RS ratio granuloma analysis 7

Supplementary Fig. 3. Histology and statistical analysis of ISH RS ratio results 8

Supplementary Fig. 4. Single-bacillus images of pre-rRNA and 23S rRNA ISH staining 9

Supplementary Fig. 5. Effect of individual drugs on ITS1/23S ratio and emergence of resistance 10

Supplementary Table 1. Statistical results from individual drug evaluation *in vitro* 11

Supplementary Fig 6. DNA and RNA synthesis continue during exposure to non-sterilizing drugs 12

Supplementary Table 2. Statistical results from evaluation of regimens in BALB/c mice 15

Supplementary Fig. 9. RS ratio during treatment with four regimens in BALB/c mice 16

Supplementary Fig. 10. RS ratio at end of treatment and relapse assessment in BALB/c mice 17

Supplementary Fig. 11. Results of confirmatory BALB/c mouse relapse study 18

Supplementary Table 3. Primer sequences & information. 19

Supplementary Methods

RNA extraction from *in vitro* samples

In vitro cultures were incubated for 5 minutes in guanidine thiocyanate RNA stabilization solution (5 M guanidine thiocyanate (Biosynth), 0.25 M sodium citrate, 1% Tween 80, 1% β-mercaptoethanol) to stabilize RNA profiles and bacilli were collected by 5-minute centrifugation at 3220×g using a tabletop centrifuge (Eppendorf 5810 R). Supernatants were discarded and cell pellets immediately suspended in Trizol (Invitrogen). Cells were disrupted using 0.1 mm diameter zirconia/silica beads (BioSpec) with three 40 second pulses in a bead beater. Samples were kept on ice for five minutes between pulses. Cellular debris was separated from the cell lysate by centrifugation for 1 minute at 21,000×g. Lysates were transferred to a new tube containing heavy phase lock gel and 300 µl chloroform, mixed vigorously for 15 seconds, then incubated at room temperature for 2 minutes with occasional mixing. The aqueous phase was removed to a separate tube for RNA extraction and mixed with 270 µl of isopropanol, then 265 µl of high salt solution (0.8 M sodium citrate, 1.2 M sodium chloride) was added and mixed by inversion. RNA was precipitated overnight at 4°C and then pelleted by centrifugation at 4°C, 21,000×g for 10 minutes. The RNA pellet was washed twice with 70% ethanol then resuspended in 88 µl nuclease-free water. 2 µl DNase I and 10 µl DNase I buffer (Roche) were added to the resuspended pellet and incubated for 30 minutes at 37°C. Following DNase I treatment, samples were purified using the RNeasy column purification (Qiagen) according to manufacturer instructions with an elution volume of 88 µl in nuclease-free water. One further DNase I treatment and RNeasy column purification were performed, with a final elution volume of 40 µl.

RNA extraction from murine samples

RNA isolation from BALB/c chronic and relapsing model samples was performed as detailed in the *in vitro* RNA extraction section with the following modifications. Lung tissue was suspended in 2 ml Trizol and tissue and bacilli disruption was achieved by 4 bead beating pulses interspaced with incubation on ice. After overnight precipitation, RNA from *in vivo* samples was purified by RNeasy column prior to DNase I treatment.

RNA isolation from the BALB/c high dose aerosol relapse study samples were bead beaten in 2 ml Trizol using the CKmix50 lysis kit for 1×16 seconds at 7,200 rpm followed by 3×30 seconds at 6,500 rpm with 5 minute rests on wet ice between cycles using the CK01 lysis kit (Bertin). RNA extraction was performed from this point as described above with the following modifications. The RNA pellet was air dried for 10 minutes at room temperature, resuspended in 80 µl water, and reconstituted for one hour on ice. RNA was then purified with the Maxwell RSC simplyRNA tissue kit using the Maxwell RSC instrument (Promega) following the manufacturer's instructions with the following modifications. An additional DNase treatment was performed with Promega RQ1 DNase prior to instrument use and additional kit DNase was added at twice the recommended amount for instrument use.

RNA extraction from human samples

All human studies used the same method of immediate RNA preservation. Briefly, spontaneously expectorated sputum was preserved within 5 minutes in guanidine thiocyanate solution. Sputa were needle sheared, centrifuged at 9000×g, and stored in Trizol at −80°C. To extract total RNA, tubes were thawed on ice and the supernatant was transferred to a tube with

0.1 mm silica beads. Cells were disrupted by bead beating three times at 30 seconds at 6 M/S (Fastprep24 bead beater, MP Biomedicals), resting on ice for 1 min in between bead beating cycles. Lysates were centrifuged 20,800×g for 1 minute and the supernatant was transferred to a new tube with chloroform and mixed. Tubes were centrifuged for 10 min at 20,800×g and the aqueous phase was transferred to a new tube with 0.1 volume 5 M ammonium acetate and equal volume of isopropanol. RNA was precipitated overnight at -20°C, followed by 10 minutes of centrifugation at 4°C at 20,800×g. The isopropanol was aspirated and the pellet was washed twice in 500 µl 70% ethanol, centrifuging at 20,800×g for 5 minutes after each wash. The pellet was air dried for 10 minutes at room temperature and resuspended in 80 µl nuclease-free water. The RNA was purified by three rounds of the RNeasy® mini column system (Qiagen) and two rounds of off-column Promega RQ1 DNase treatments.

Nucleic acid radiolabeling

Uracil, [6-3H] (Moravek Biochemicals) was added to cultures at the time of drug addition at a final concentration of 1 µCi ml^{-1} for labeling windows of 12 hours for control cultures and 24 hours for drug-treated cultures. Nucleic acid extraction was performed as described above with the following modifications. No heavy phase lock gel was used and 200 µl chloroform was used. For RNA, only one DNase I treatment and RNeasy column purification were performed with a final elution volume of 100 µl. To extract DNA, 500 µl of back extraction buffer (4 M guanidine thiocyanate, 50 mM sodium citrate, 1 M Tris base) was added to the organic phase of the nucleic acid Trizol/chloroform extraction and samples were incubated for 10 minutes at room temperature. Centrifugation of the samples was performed at 21,000×g for 30 minutes to separate organic and aqueous phases. The aqueous phase of each sample was placed in a new

tube, 10 µl glycoblue (Invitrogen) and 400 µl of isopropanol were added. Samples were mixed by inversion for 10 minutes at room temperature. DNA was pelleted by centrifugation at 21,000×g for 15 minutes at 4°C and the pellet was washed twice with 70% ethanol. The pellet was dried and resuspended in 100 µl nuclease-free water. 90 µl of purified nucleic acid was mixed with 5 ml Econo-Safe scintillation fluid (Grainger) and analyzed using a LS 6500 scintillation counter (Beckman Coulter).

Ethics statement and institutional review boards

This manuscript includes three human studies. All participants in these three studies provided written informed consent for the use of their sputum and clinical information to develop new biomarkers measuring the effectiveness of treatment. The institutional review boards that supervised these studies are listed below.

**Cohort**	**Study Location**	**Supervising Institutional Review Boards**
Study 36: A Platform for Assessment of TB Treatment Outcomes An Observational Study of Individuals Treated for Pulmonary Tuberculosis	Vietnam	1. Vietnam Ministry of Health Ethical Committee in National Biological Medical Research. Protocol 12/QD-BYT<br>2. University of California San Francisco Human Research Protection Program Institutional Review Board. Protocol H8660-27882-06<br>3. US Centers for Disease Control and Prevention Institutional Review Board 2. Protocol 6560.0
J Infect Dis 212, 990-998 (2015)	Uganda	1. Uganda National Council for Science and Technology. Protocol HS 259<br>2. Makerere University Faculty of Medicine Research and Ethics Committee. Protocol 2006-017<br>3. Mulago Hospital Institutional Review Board. Protocol 2006-017<br>4. Colorado Multiple Institutions Review Board. Protocol 10-0290<br>5. University of California San Francisco Human Research Protection Program Institutional Review Board. Protocol H8660-27882
RAFA Study PACTR201105000291300	Benin	1. National Ethics Committee for Research in Health, Benin. Protocol 004 31 March 2011<br>2. University of Cape Town Institutional Review Board. Protocol 153/2011<br>3. London School of Hygiene and Tropical Medicine Ethics Committee. Protocol 5917

Supplementary Results

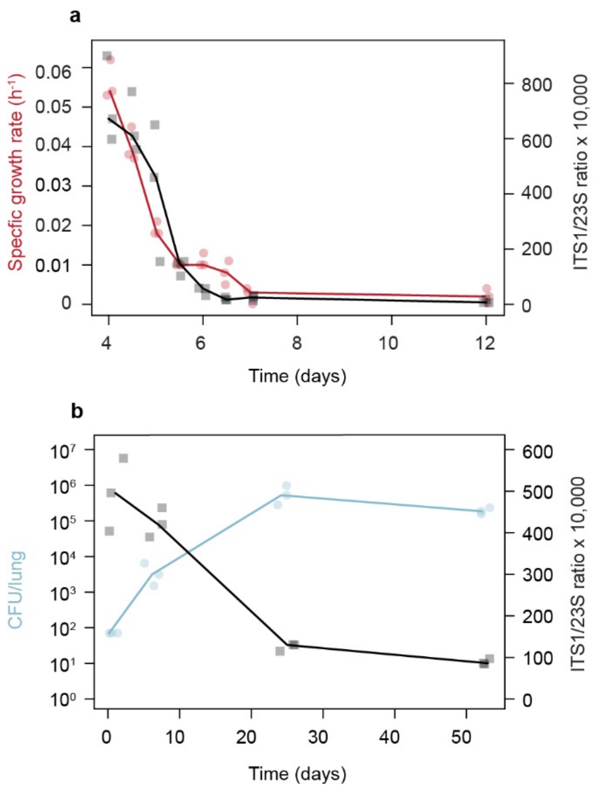

Supplementary Fig. 1. RS ratio correlates with *Mtb* replication as measured by ITS1/23S ratio

a, An additional pre-rRNA target (ITS1) recapitulated results with the RS ratio (based on ETS1). As observed with the RS ratio, ITS1/23S x 10^4 (black) mirored specific growth rate (red) in an *in vitro* progressive oxygen depletion model. Dots represent values from three independent experiments. Lines connect median values **b**, ITS1/23S also recapitulated RS ratio results in the BALB/c chronic infection model. ITS1/23S x 10^4 (black) indicated rapid initial rRNA synthesis that slowed as *Mtb* burden (blue) plateaued. Dots represent values from three individual mice. Lines connect mean values from each timepoint.

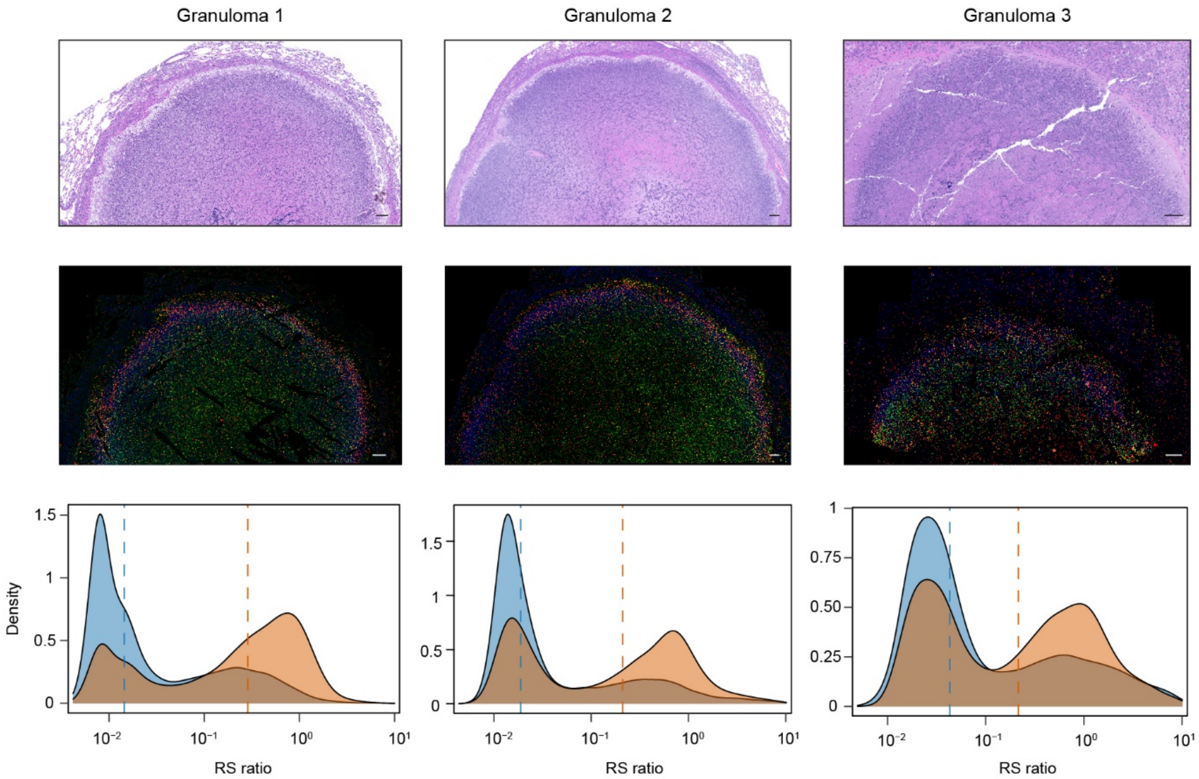

Supplementary Fig. 2. Replicate results for ISH RS ratio granuloma analysis

H&E staining of granulomas from untreated mice infected in three separate experiments (top row). Nearby tissue sections (middle row) were labeled by ISH for pre-rRNA (red) and 23S-rRNA (green), and DAPI (host-cell nuclei, blue). Granuloma 1 serves as a technical replicate of the section analyzed in Fig. 2 of the manuscript. Granulomas 2 and 3 are biological replicates from animals infected in separate experiments. The density plots (bottom row) depict the distribution of the RS ratio in the inflammatory rim (orange) compared to the caseum (blue), and the population of overlapping bacteria (brown). The median RS ratio for each population is represented by a dashed vertical line. For each granuloma, the RS ratio was significantly higher in the inflammatory rim as evaluated by the two-sided, two-sample Kolmogorov-Smirnov test to measure the difference between the two distributions (*P*-values in each granuloma < 2e-16). Images are magnified to 40X and scale bars represent 100 µm.

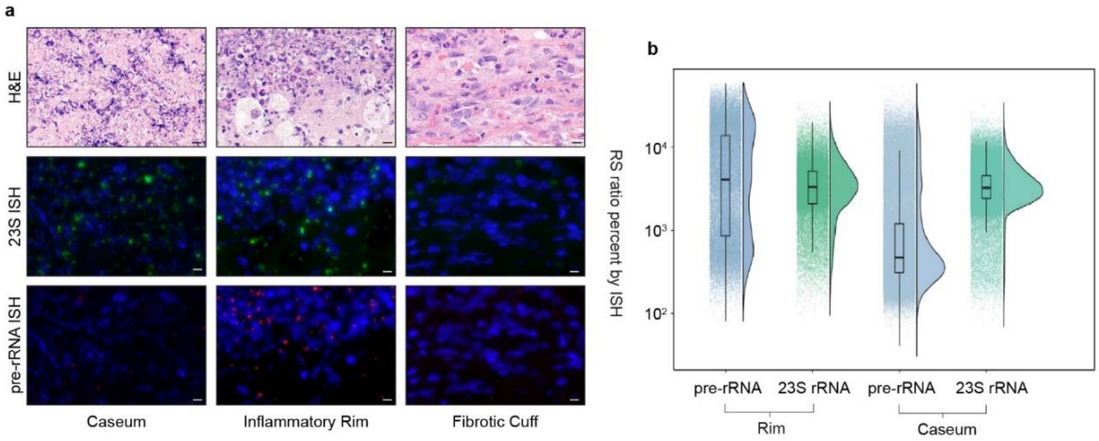

c

Region	Number of bacilli counted	Bacilli/µm²	RS ratio (variance)	pre-rRNA median MFI (variance)	23S rRNA median MFI (variance)	Variance pre-rRNA vs 23S rRNA *p*-value
Rim	38,305	39.3	1.03 (0.64)	3.6 (0.28)	3.52 (0.06)	< 0.0001
Caseum	126,573	37.7	0.13 (0.38)	2.67 (0.43)	3.5 (0.13)	< 0.0001
p-value Rim vs Caseum	-	-	< 0.0001 (< 0.0001)	< 0.0001	0.62	-

Supplementary Fig. 3. Histology and statistical analysis of ISH RS ratio results

a, Representative high magnification images of the three distinct regions of the single type 1 granuloma shown in Fig. 2 of the main manuscript. Region 1 (Caseum) consists entirely of necrotic cellular debris without cytological detail. Region 2 (Inflammatory Rim) details the intersecting infiltration of viable and degenerate neutrophils and highly vacuolated macrophages. Region 3 (Fibrotic Cuff) depicts the compressed alveolar lung tissue with fibrosis and few infiltrating leukocytes. Images from each region in two nearby tissue sections are shown. One tissue section was stained using hematoxylin and eosin (H&E) and the other by multiplexed ISH for 23S rRNA (green, middle row) and pre-rRNA (red, bottom row) with DAPI (host-cell nuclei, blue). Images are magnified to 400X and scale bars represent 5 µm. **b,** Population distribution of 164,878 individual bacillus Mean Fluorescence Intensity (MFI) from ISH for pre-rRNA and 23S rRNA in a single granuloma. These MFI values represent the pre-rRNA numerator (blue) and 23S rRNA denominator (green) of the ISH RS ratio results displayed in Fig 2e of the manuscript. Components of this raincloud plot are: (1) density plots for the distribution of RS ratio values on a $\log_{10}$ scale, (2) scatterplots to visualize all points measured, and (3) boxplots to present the range of values in the RS ratio. The center and ends of the box represent the median and first and third quartiles of the RS ratio. The boxplot whiskers represent the maximum and minimum values in each group. **c,** Characteristics of the *Mtb* population in the inflammatory rim and the caseum. The ratio of pre-rRNA and 23S-rRNA MFI in $\log_{10}$ values were tested for differences in dominance of median values between locations. Differences in measured ranges of MFI values were calculated by variance. The ratio of pre-rRNA to 23S rRNA MFI was significantly higher in the inflammatory rim than in the caseum, consistent with slower rRNA synthesis and replication in the caseum. Pre-rRNA MFI was significantly higher in the inflammatory rim while 23S MFI was similar in the rim and caseum. A two-sided F-test was used to compare the variance between anatomical regions. Comparing the variance of the ratio of pre-rRNA and 23S for individual bacilli indicated significantly greater heterogeneity in the population of bacilli in the rim compared to the caseum (*P*-values < 2e-16). *P*-values <0.05 are indicative of statistical significance.

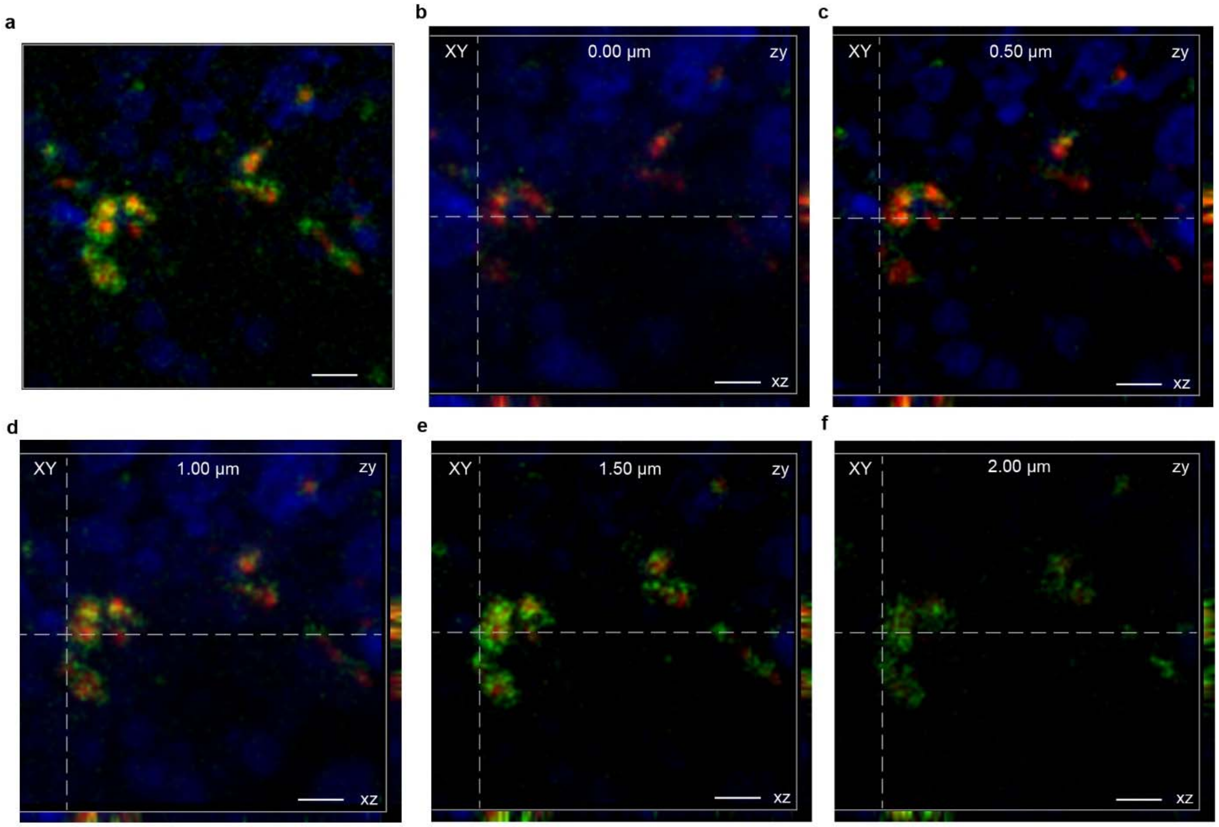

Supplementary Fig. 4. Single-bacillus images of pre-rRNA and 23S rRNA ISH staining

a, High-magnification overlay of multiplexed ISH from the single image in Fig. 2g of the manuscript is enlarged to show the distribution of 23S-rRNA (green), pre-rRNA (red) in individual bacilli. Host-cell nuclei are stained with DAPI (blue). **b-f,** Individual images from Z-stacked frames taken 0.5 µm apart allow visualization of the spatial relationship of rRNA signals within individual bacilli across X,Y, and Z planes. Together these images confirm the distribution of 23S signals in a reticular pattern around a central confluence of pre-rRNA signals. The scale bar indicates 5 µm.

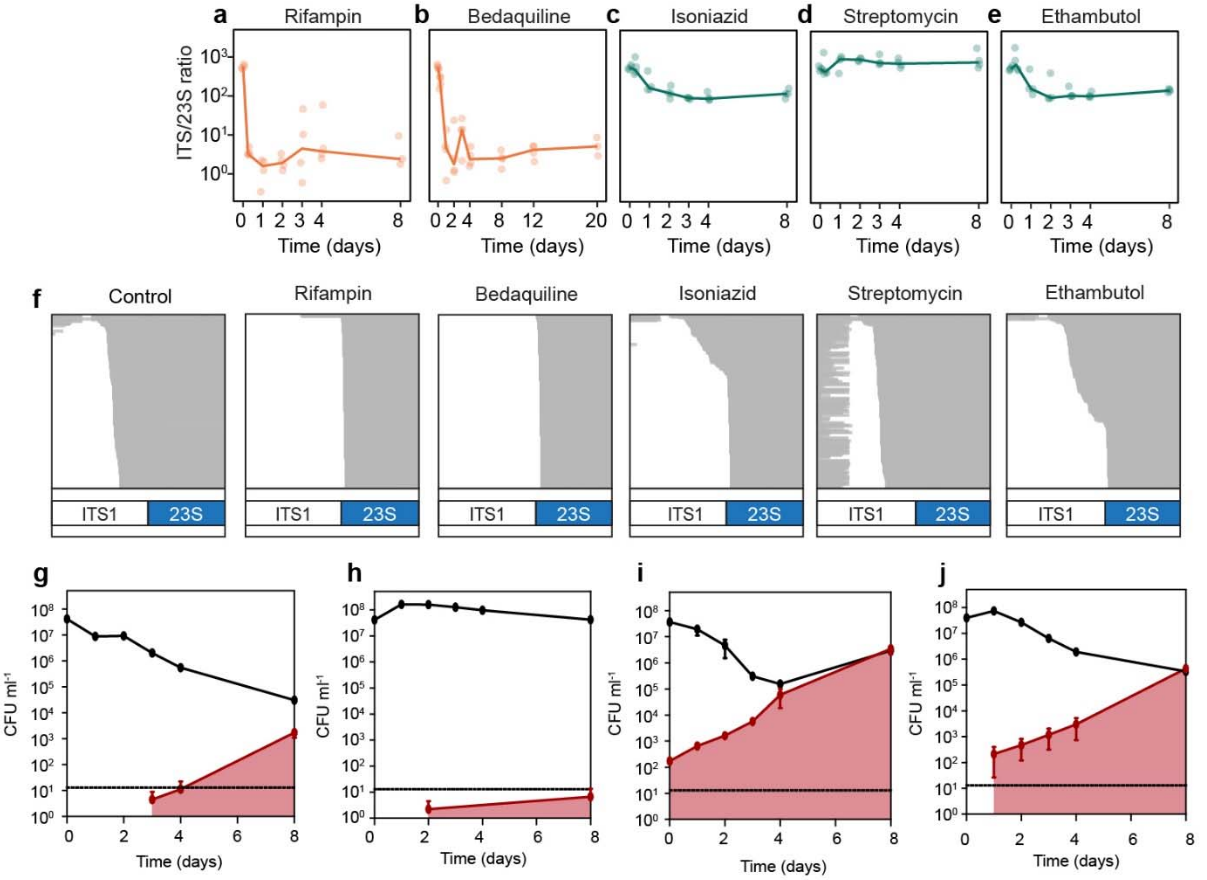

Supplementary Fig. 5. Effect of individual drugs on ITS1/23S ratio and emergence of resistance

a-e, Change in ITS1/23S x 10^4 in time kill-experiments recapitulated RS ratio results for rifampin, bedaquiline, isoniazid, streptomycin and ethambutol. Dots represent values from three independent experiments. Lines connect mean values from each time point. Sterilizing and non-sterilizing drugs are shown in orange and green, respectively. **f**, Integrative Genomics Viewer (IGV) images of RNAseq data summarize expression of the ITS1 region for log-phase untreated control, rifampin, bedaquiline, isoniazid, streptomycin, and ethambutol. Gray shading denotes individual reads. White and blue bars indicate the ITS1 pre-rRNA and 23S rRNA regions, respectively. **g-j,** Drug-resistant populations *in vitro*. Total plateable bacteria (black lines) and resistant bacteria (red lines with fill) over time in cultures treated with **g,** rifampin **h,** bedaquiline **i,** isoniazid, and **j,** streptomycin. Ethambutol resistance was not discernable. Error bars represent standard error of the mean of three independent experiments. The dashed line represents the limit of detection (13 CFU ml^{-1}).

Supplementary Table 1. Statistical results from individual drug evaluation *in vitro*

Pairwise comparisons between all drugs were performed using the Kruskal-Wallis one-way analysis of variance. Fold change indicates median fold decrease in RS ratio versus untreated control at the onset of drug treatment. Data were analyzed from three independent experiments.

		RIF	BDQ	INH	STR	EMB
**6 hours**	Fold change‡	131	6.08	0.715	0.414	0.624
RIF	131	-	0.0394	0.0001	0.0000	0.0000
BDQ	6.08		-	0.0039	0.0002	0.0009
INH	0.715			-	0.0889	0.2452
STR	0.414				-	0.2452
EMB						-
**24 hours**	Fold change	452	137	1.41	0.181	1.55
RIF	452	-	0.0346	0.0000	0.0000	0.0000
BDQ	137		-	0.0003	0.0000	0.0005
INH	1.41			-	0.0010	0.3744
STR	0.181				-	0.0005
EMB	1.55					-
**48 hours**	Fold change	546	257	2.89	0.191	2.05
RIF	546	-	0.0637	0.0000	0.0000	0.0000
BDQ	257		-	0.0008	0.0000	0.0001
INH	2.89			-	0.0003	0.1741
STR	0.191				-	0.0020
EMB	2.05					-

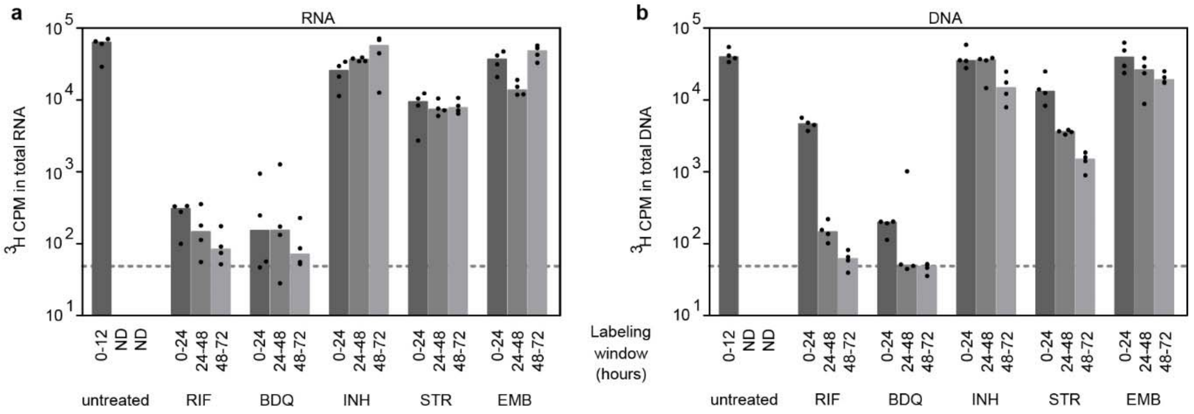

Supplementary Fig 6. DNA and RNA synthesis continue during exposure to non-sterilizing drugs

De novo cellular **a,** RNA and **b,** DNA synthesis following addition of sterilizing and non-sterilizing drugs. Bars indicate new RNA and DNA synthesis assessed by accumulation of ^{3}H-labeled nucleotides into the total pool of each nucleic acid in three independent experiments. ^{3}H-labeling windows following drug addition are indicated beneath each bar. Total ^{3}H counts per minute (CPM) in total RNA or DNA pool for each culture are shown on the y-axis. The dashed line indicates the limit of detection (defined as CPM >2-fold above background CPM).

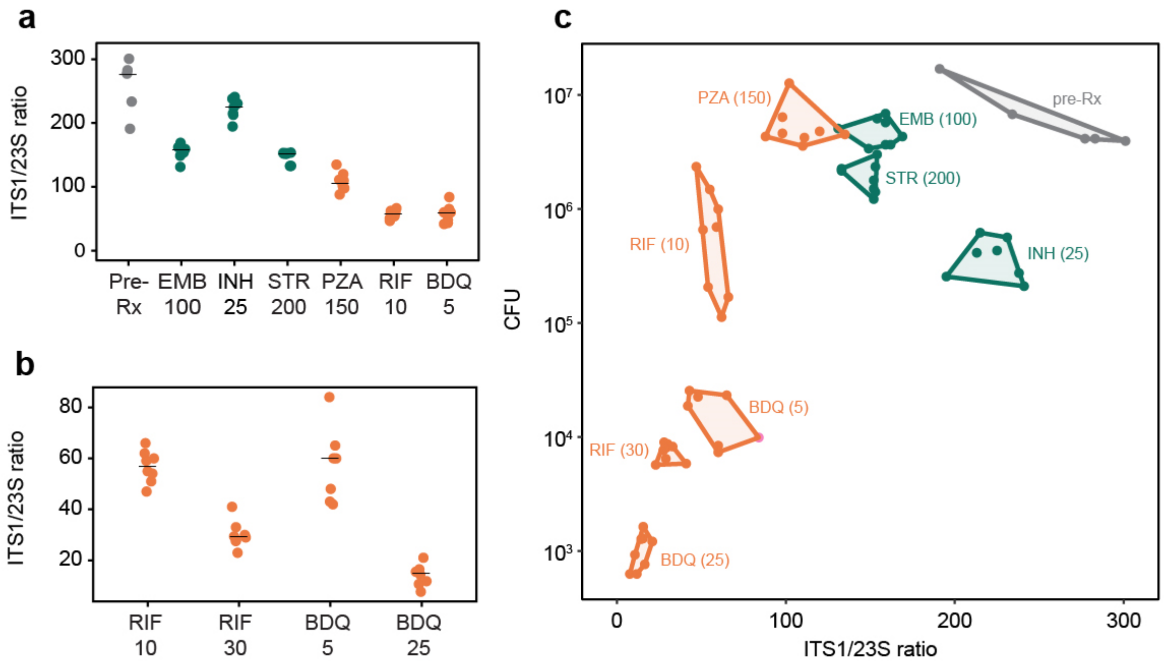

Supplementary Fig. 7. Effect of sterilizing and non-sterilizing drugs on ITS1/23S in mice

An additional pre-rRNA target (ITS1) recapitulated results with the RS ratio (based on ETS1). **a,** ITS1/23S x 10^4 in lung tissue of BALB/c mice treated for 28 days with individual non-sterilizing (green) and sterilizing (orange) drugs. Dots indicate individual mice (N=8 per group). Horizontal lines indicate group means. **b,** Dose-response relationship between rifampin and bedaquiline dose and ITS1/23S x 10^4. Dots indicate individual mice (N=8 per group). Horizontal lines indicate group means. **c,** Contrasting effects of 28-day treatment with individual drugs/doses on CFU and ITS1/23S x 10^4 in BALB/c mice. A convex hull was used to display data groupings.

a

Group	Log10 drug-resistant CFU per lung (number of animals with detectable resistance/total)	
	Start of Treatment	End of Treatment
Rifampin (10 mg/kg)	< 2.18 (0/5)	< 2.18 (0/8)
Rifampin (30 mg/kg)	< 2.18 (0/5)	< 2.18 (0/8)
Bedaquiline (5 mg/kg)	< 2.18 (0/5)	2.18 (1/8)
Bedaquiline (25 mg/kg)	< 2.18 (0/5)	nd
Isoniazid (25 mg/kg)	< 2.18 (0/5)	< 2.18 (0/8)
Streptomycin (200 mg/kg)	2.36 (0/5)	3.15 (8/8)

CFU of < 2.18 indicates the lower level of detection; nd not determined

b

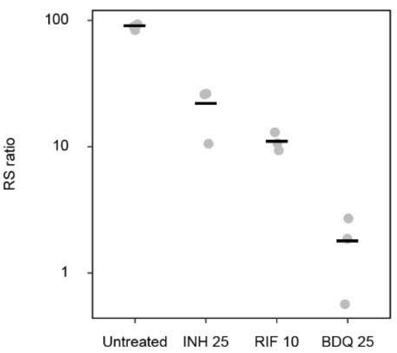

c

Drug Treatment	Control	EMB (100)	INH (25)	STR (200)	PZA (150)	RIF (10)	RIF (30)	BDQ (5)	BDQ (25)	PZA (150) + RIF (10)
Control	-	0.0016	0.0025	0.0016	0.0016	0.0016	0.0016	0.0025	0.0016	0.0016
EMB (100)	2.4	-	0.4634	0.3823	0.0002	0.0002	0.0002	0.0003	0.0002	0.0002
INH (25)	2.1	0.9	-	0.7789	0.0003	0.0003	0.0003	0.0006	0.0003	0.0003
STR (200)	2.1	0.9	1.0	-	0.0002	0.0002	0.0002	0.0003	0.0002	0.0002
PZA (150)	4.4	1.9	2.0	2.1	-	0.0002	0.0002	0.0059	0.0002	0.0002
RIF (10)	11.6	5.0	5.3	5.4	2.6	-	0.0003	0.0003	0.0002	0.0003
RIF (30)	18.1	7.8	8.3	8.4	4.1	1.5	-	0.0003	0.0650	0.4418
BDQ (5)	7.8	3.4	3.6	3.7	1.8	0.7	0.4	-	0.0003	0.0003
BDQ (25)	27.4	11.9	12.5	12.8	6.2	2.3	1.5	3.8	-	0.0830
PZA (150) + RIF (10)	20.1	8.7	9.2	9.4	4.5	1.7	1.1	2.8	0.8	-

Supplementary Fig. 8. Resistance and statistical results for individual drugs in BALB/c mice

a, Drug resistant CFU at the start and end of 28-day, single-drug treatment in the BALB/c mouse high-dose aerosol TB efficacy model. CFU of <2.18 indicates the lower level of detection; nd indicates not determined. **b,** RS ratio following 28 days of treatment with single drugs compared with untreated day 53 control in the low-dose aerosol BALB/c model (N=3 mice per drug). Circles represent values from individual mice. Horizontal bars indicate the group median. **c,** Pairwise fold-change and two-sided, two-sample Wilcoxon test *P*-values between BALB/c mice treated with individual drugs or the combination of PZA and RIF in the BALB/c high-dose aerosol model presented in Fig. 4 of the manuscript. Fold-changes are shown in the lower left-hand quadrant. *P*-values are shown in the upper right-hand quadrant.

Supplementary Table 2. Statistical results from evaluation of regimens in BALB/c mice

Pairwise *P*-values using two-sided Fisher's exact test for relapse proportions between timepoints and regimens tested in the BALB/c relapsing mouse experiment summarized in Fig. 5 of the manuscript.

		HRZE			PaMZ			BPaL			BPaMZ		
	Time (weeks)	14 weeks	16 weeks	20 weeks	12 weeks	16 weeks	20 weeks	4 weeks	8 weeks	12 weeks	4 weeks	6 weeks	8 weeks
HRZE	14 weeks	-	0.2241	<0.0001	<0.0001	<0.0001	<0.0001	>0.9999	0.0169	<0.0001	0.4828	<0.0001	<0.0001
	16 weeks		-	0.0007	0.0028	<0.0001	<0.0001	0.2241	0.427	<0.0001	>0.9999	0.0001	<0.0001
	20 weeks			-	>0.9999	0.4828	0.4828	<0.0001	0.0209	0.4828	0.0001	>0.9999	0.4828
PaMZ	12 weeks				-	0.2241	0.2241	<0.0001	0.0604	0.2241	0.0007	0.5977	0.2241
	16 weeks					-	>0.9999	<0.0001	0.0007	>0.9999	<0.0001	>0.9999	>0.9999
	20 weeks						-	<0.0001	0.0007	>0.9999	<0.0001	>0.9999	>0.9999
BPaL	4 weeks							-	0.0169	<0.0001	0.4828	<0.0001	<0.0001
	8 weeks								-	0.0007	0.2148	0.0052	0.0007
	12 weeks									-	<0.0001	>0.9999	>0.9999
BPaMZ	4 weeks										-	<0.0001	<0.0001
	6 weeks											-	>0.9999
	8 weeks												-

a

Regimen	Test of Trend p-value
BPaMZ	0.004
BPaL	<0.0001
PaMZ	0.008
HRZE	0.001

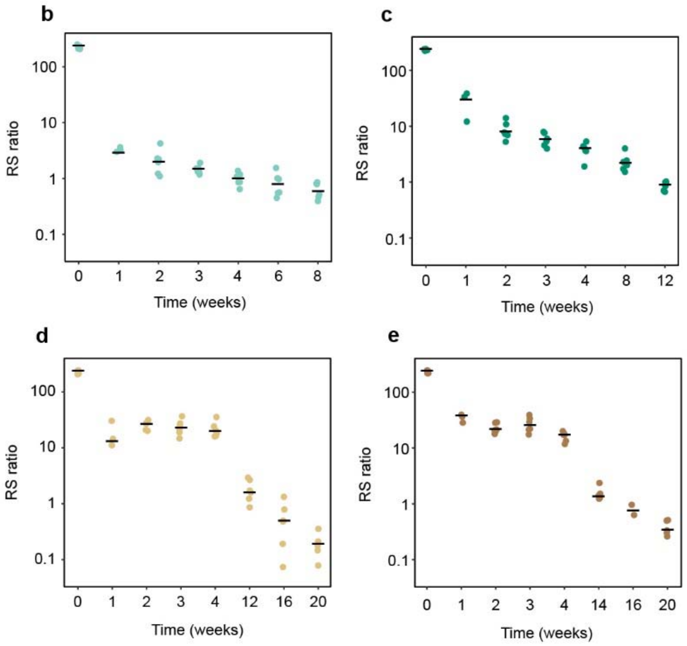

Supplementary Fig. 9. RS ratio during treatment with four regimens in BALB/c mice

a, Trend test *P*-value using 1 sample two-sided Mann-Kendall Test without adjustment for multiple comparisons for the relationship between treatment duration and RS ratio for each regimen tested in the BALB/c relapse experiment. **b-e,** change in RS ratio during the entire treatment course for BPaMZ (light green), BPaL (dark green), PaMZ (light brown), and HRZE (dark brown). Circles represent individual mice (N=3 per regimen at week 1 and N=6 per regimen at all additional timepoints). Horizontal bars represent group median values.

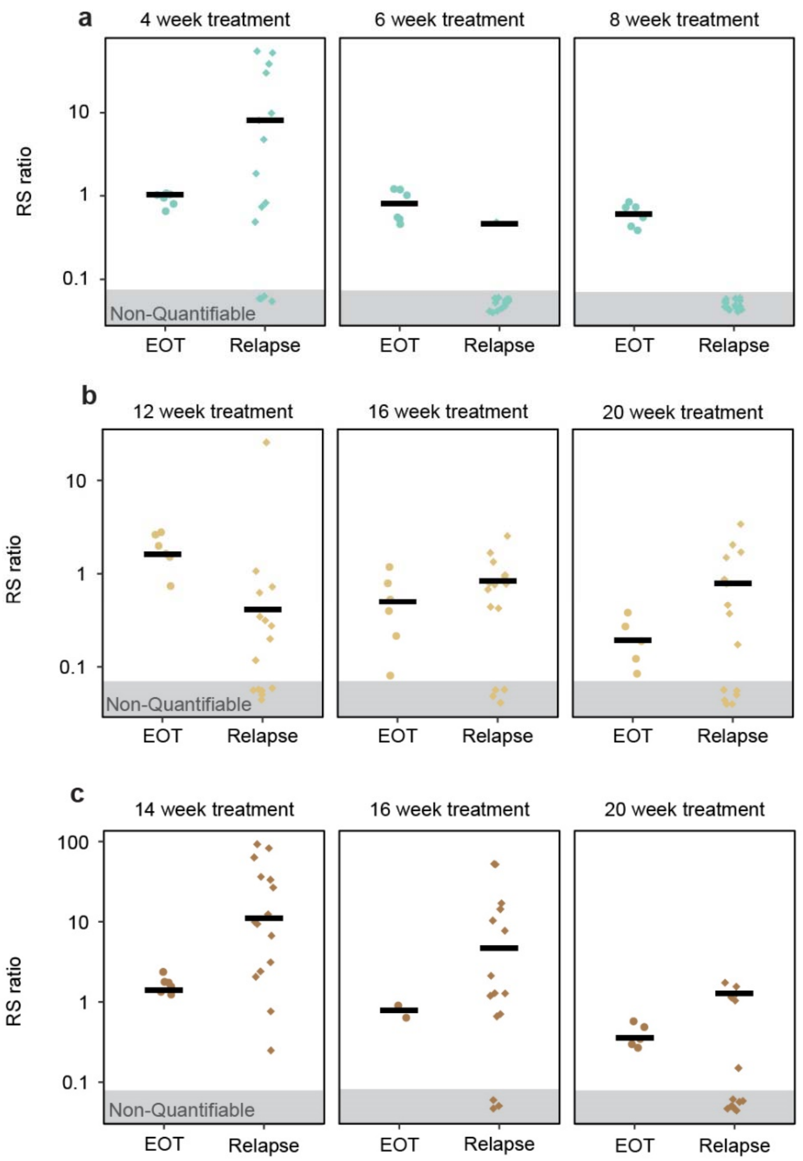

Supplementary Fig. 10. RS ratio at end of treatment and relapse assessment in BALB/c mice

a-c, RS ratio results following three treatment durations with BPaMZ (light green), PaMZ (light brown), and HRZE (dark brown). Circles represent individual mice at end of treatment (EOT) (N=6 per regimen and duration). Squares represent mice at time of relapse determination after a 12-week drug holiday (N=15 per regimen and duration). Horizontal bars represent group median values.

a

	Mean ± SEM log_{10} CFU (n/n = mice with CFU/total)		
Drug Regimen	Pretreatment	4 Weeks treatment	8 weeks treatment
Untreated	7.42 ± 0.06 (6/6)	-	-
HRZE	-	4.03 ± 0.07 (6/6)	1.38 ± 0.12 (6/6)
BPaL	-	2.51 ± 0.24 (4*/6)	< 0.88 ± 0.0 (0/6)

*plate counts lost due to contamination

b **c**

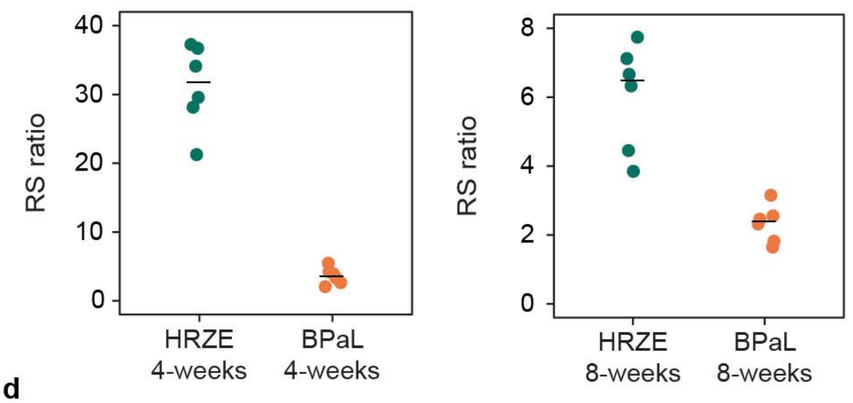

d

	Proportion of mice relapsing 12 weeks after treatment completetion (number relapsed/number tested (%))		
Regimen administered	8 weeks treatment	12 weeks treatment	16 weeks treatment
HRZE	nd	15/15 (100%)	11/13 (85%)
BPaL	4/14 (29%)	0/13 (0%)	nd

nd, not determined

Supplementary Fig. 11. Results of confirmatory BALB/c mouse relapse study

Results of additional independent BALB/c relapse study, separate from data presented in Fig. 5 of the manuscript. **a,** CFU burden in lung homogenate of BALB/c mice treated for 4 or 8 weeks with HRZE or BPaL **b-c,** RS ratios in lung homogenate of BALB/c mice treated for **b,** 4 weeks or **c,** 8 weeks of HRZE (green) or BPaL (orange). Circles represent individual mice (N=6 per regimen and time point). Horizontal bars represent group median values **d,** Proportion of mice with microbiologic relapse after variable treatment durations followed by a standard 12-week drug-free observation period in the BALB/c relapsing mouse model.

Supplementary Table 3. Primer sequences & information.

Primer/Probe name	Sequence (5'-3')	Fluorescent dye (5')	Modification (3')
ETS1 Forward	CCGTTTGTTTTGTCAGGATATTTCT		
ETS1 Probe	AATACCTTTGGCTCCCTTT	FAM	MGB
ETS1 Reverse	CAAAACCCAAACACTCCCTTTG		
ITS1 Forward	GGTGTGGTGTTTGAGAACTGGAT		
ITS1 Probe	TGGTTGCGAGCATC	FAM	MGB
ITS1 Reverse	GCTAGCCGGCAGCGTATC		
23S Forward	GCAGCGAAAGCGAGTCTGA		
23S Probe	AGGGCGACCCACACGCGC	HEX	ZEN (internal), IBFQ
23S Reverse	CCAGAACACGCCACTATTCACA		
