# Supplementary material for: Mycobacterium tuberculosis precursor rRNA as a measure of treatment-shortening activity of drugs and regimens
Source: Nat Commun. 2021 May 18;12:2899. doi: 10.1038/s41467-021-22833-6 (PMC8131613; doi:10.1038/s41467-021-22833-6)
Supplement: Supplementary file 3 — Reporting Summary [file 41467_2021_22833_MOESM3_ESM.pdf]

nature research

Corresponding author(s): Nicholas D Walter
Martin I Voskuil

Last updated by author(s): Mar 5, 2021

Reporting Summary

Nature Research wishes to improve the reproducibility of the work that we publish. This form provides structure for consistency and transparency in reporting. For further information on Nature Research policies, seeAuthors & Referees and theEditorial Policy Checklist .

Statistics

For all statistical analyses, confirm that the following items are present in the figure legend, table legend, main text, or Methods section.

n/a	Confirmed	
[ ]	[x]	The exact sample size (*n*) for each experimental group/condition, given as a discrete number and unit of measurement
[ ]	[x]	A statement on whether measurements were taken from distinct samples or whether the same sample was measured repeatedly
[ ]	[x]	The statistical test(s) used AND whether they are one- or two-sided<br>*Only common tests should be described solely by name; describe more complex techniques in the Methods section.*
[x]	[ ]	A description of all covariates tested
[x]	[ ]	A description of any assumptions or corrections, such as tests of normality and adjustment for multiple comparisons
[ ]	[x]	A full description of the statistical parameters including central tendency (e.g. means) or other basic estimates (e.g. regression coefficient) AND variation (e.g. standard deviation) or associated estimates of uncertainty (e.g. confidence intervals)
[ ]	[x]	For null hypothesis testing, the test statistic (e.g. *F*, *t*, *r*) with confidence intervals, effect sizes, degrees of freedom and *P* value noted<br>*Give P values as exact values whenever suitable.*
[x]	[ ]	For Bayesian analysis, information on the choice of priors and Markov chain Monte Carlo settings
[x]	[ ]	For hierarchical and complex designs, identification of the appropriate level for tests and full reporting of outcomes
[x]	[ ]	Estimates of effect sizes (e.g. Cohen's *d*, Pearson's *r*), indicating how they were calculated

Our web collection on statistics for biologists contains articles on many of the points above.

Software and code

Policy information about availability of computer code

Data collection	The following software packages were used for data collection:<br>Collection of droplet digital PCR data used the QX200 Droplet Digital PCR system software QuantiSoft Analysis Pro v1.0. RNA sequencing pre-processing was accomplished using BBTools v 35.85 (https://sourceforge.net/projects/bbmap/) and Bowtie2 v2.4.1 (http://bowtie-bio.sourceforge.net/bowtie2/index.shtml). For in situ hybridization, image analysis was performed using the ilastik machine-learning-based (bio)image analysis v1.3.3 (www.ilastik.org). The ilastik plugin for ImageJ (v. 2.1.0/1.53c) was used to export image data.
Data analysis	Graphical representations of RNA sequence data were created using Integrative Genomics Viewer v2.8 (http://software.broadinstitute.org/software/igv/). The population pharmacokinetic model for rifampin was developed using nonlinear mixed-effects modeling in software NONMEM v7.3 (Icon Development Solutions). Certain graphics were generated using SigmaPlot software v 11 (SYSTAT). Additional analyses were performed using R software v3.5.2 (R Foundation for Statistical Computing, www.R-project.org).

For manuscripts utilizing custom algorithms or software that are central to the research but not yet described in published literature, software must be made available to editors/reviewers. We strongly encourage code deposition in a community repository (e.g. GitHub). See the Nature Research guidelines for submitting code & software for further information.

Data

Policy information about availability of data

All manuscripts must include a data availability statement. This statement should provide the following information, where applicable:

- Accession codes, unique identifiers, or web links for publicly available datasets
- A list of figures that have associated raw data
- A description of any restrictions on data availability

All raw sequencing data have been deposited in the Sequence Read Archive (SRA) under BioProject accession PRJNA615137. Individual samples have the following BioSample accession numbers: untreated, SAMN14446914; rifampin, SAMN14446915; isoniazid, SAMN14446916; streptomycin, SAMN14446917; ethambutol, SAMN14446918; bedaquiline, SAMN14446919. All other data supporting the findings of this study are available within the paper and its supplementary information

files.

Field-specific reporting

Please select the one below that is the best fit for your research. If you are not sure, read the appropriate sections before making your selection.

- [x] Life sciences
- [ ] Behavioural & social sciences
- [ ] Ecological, evolutionary & environmental sciences

For a reference copy of the document with all sections, see nature.com/documents/nr-reporting-summary-flat.pdf

Life sciences study design

All studies must disclose on these points even when the disclosure is negative.

Sample size

For in vitro experiments, no sample size calculation was performed. We chose arbitrarily to conduct four replicate biological experiments. The variation between replicate experiments was low and the effect size differentiating drug arms was large so we concluded that additional replicate experiments with the same drugs was not necessary. For murine experiments, we performed a sample size calculation based on preliminary data (not presented) which indicated N=8 for each drug arm. Our murine results show low variation between mice and a large effect size between different drugs which provides reassurance that the experiment was adequately powered. The human studies were exploratory and we lacked sufficient preliminary data to perform a power analysis. However, as in the in vitro studies, the effect size was large and the number of observation appears sufficient to support our conclusions.

Data exclusions

No data were excluded from analysis.

Replication

Both the in vitro and murine studies include a number of biological replicates. Variation between biological replicates is described in the manuscript. It was not feasible to repeat the human studies. However, observations in the two human studies are consistent.

Randomization

For in vitro drug exposures, each independent experiment included 80-100 glass tubes, each containing 5mL Mtb culture diluted 7H9 medium. Because the tubes were standard and prepared first, irrespective of drug exposure, the assignment of a particular tube to a particular drug was random. Over the course of several minutes, drugs were added sequentially to all tubes one drug arm at a time (i.e., rifampin added to assigned tubes, then isoniazid added, etc). Since the experimental time course spanned days, lack of randomization in the addition of drugs resulted in no systematic bias. We determined that randomization at this stage would have added complexity, increasing risk of experimental error without enhancing the integrity of our results.

There was no randomization for the C3HeB/FeJ mouse because it was a single-arm study of untreated mice at a single time point. There was no factor on which to randomize. For BALB/c studies, experiments included 6- to 8-week female mice purchased and delivered as a single batch per experiment. Mice were randomly assigned into groups for infection and further distributed at random into individual treatment arms. At the initiation of the treatment, these mice were randomly assigned to a particular sacrifice time point corresponding to a treatment group number and study endpoint designation (e.g., group 1, 4 week sacrifice or group 2, 12 week relapse, etc.). Treatments and sacrifices and PD marker assessments were each performed by different technical staff to prevent any introduction of bias.

This manuscript includes three human studies. The first two studies, conducted in Vietnam and Uganda, were observational. There was no intervention and therefore no randomization. The third study, conducted in Benin was a biomarker substudy conducted in a double-blind, randomized clinical trial. Participants were randomized as a component of the parent RAFA trial. The biomarker substudy itself did not involve randomization.

Blinding

For in vitro studies, CFU counts were made without reference to group assignments. There was no blinding for the C3HeB/FeJ mouse study because it was a single-arm study of untreated mice at a single time point. For BALB/c studies, blinding was as follows. CFU was enumerated from >21 days after initial plating. CFU counts were listed by mouse and by group number, but did not include specific drug treatment arm information. Once scored, the hand written CFU counts were scanned as raw data CFU files which were later transcribed into excel worksheets revealing the the individual study treatment groups. For both in vitro and murine studies, samples were labeled with a numerical code that served as the primary identifier for the lab conducting RS ratio profiling.

This manuscript includes three human studies. The first and second studies, conducted in Vietnam and Uganda, were observational studies. There was no intervention and no factor to be blinded to. The third study, conducted in Benin was a substudy conducted in a double-blind, randomized clinical trial. In this study, investigators and staff were blinded to treatment allocation both during the parent clinical trial and in the biomarker sub-study.

Reporting for specific materials, systems and methods

We require information from authors about some types of materials, experimental systems and methods used in many studies. Here, indicate whether each material, system or method listed is relevant to your study. If you are not sure if a list item applies to your research, read the appropriate section before selecting a response.

Materials & experimental systems

n/a	Involved in the study	
x		Antibodies
x		Eukaryotic cell lines
x		Palaeontology
	x	Animals and other organisms
	x	Human research participants
x		Clinical data

Methods

n/a	Involved in the study	
x		ChIP-seq
x		Flow cytometry
x		MRI-based neuroimaging

Animals and other organisms

Policy information about studies involving animals; ARRIVE guidelines recommended for reporting animal research

Laboratory animals

This manuscript includes two mouse strains (species Mus musculus):
1. Balb/c female mice, 6-8 weeks old
2. C3HeB/FeJ female mice, 6-8 weeks old

Mice were housed socially (2-5 animals per cage) in a certified ABSL-3 facility in HEPA filter equipped techniplast cages on autoclaved bedding changed every 7-14 days. Mice had access to irradiated chow and water, ad libitum. Twelve hour light/dark cycles are employed and mice are maintained at temperatures between 65 and 75° F with 40-60% humidity.

Wild animals

The study did not involve wild animals.

Field-collected samples

The study did not involve collection of field samples.

Ethics oversight

Ethics oversight was provided by the Colorado State University Animal Care and Use Program which is PHS Assured (A3572-01), USDA Registered (84-R-0003), and AAALAC accredited (#000834). The IACUC approved CSU protocol number is 17-7701A.

Note that full information on the approval of the study protocol must also be provided in the manuscript.

Human research participants

Policy information about studies involving human research participants

Population characteristics

This manuscript includes three human studies. The first was a longitudinal study of TB patients treated under routine care, conducted across eight outpatient clinics in Hanoi, Vietnam, by the US CDC TB Trials Consortium at the UCSF/Vietnam National TB Programme network. Entitled “Study 36: An Platform for Assessment of TB Treatment Outcomes An Observational Study of Individuals Treated for Pulmonary Tuberculosis.” The second was a longitudinal observational study in Uganda that included 17 adult inpatients (male and female) treated for drug-susceptible TB per local guidelines with the global standard 4-drug regimen at standard doses. This cohort has been previously described in J Infect Dis 212, 990-998 (2015). The third was a biomarker sub-study embedded in the Benin site of the RAFA trial which enrolled adults living with HIV who were co-infected with drug susceptible TB. Patients were randomized to either a control arm, which was standard of care at the time (standard antitubercular treatment with 10 mg/kg doses of rifampicin and start of ART 8 weeks thereafter), or to early start of ART (2 weeks after initiating antituberculosis treatment), or to receive a high dose of rifampicin in the first 8 weeks of TB treatment (50% dose increase, i.e. 15 mg/kg, and start of ART 8 weeks after initiating antituberculosis treatment). The RAFA trial has previously been described in J Antimicrob Chemother 74, 139–148 (2019).

Recruitment

The Vietnam study was a random sample of consecutive adults diagnosed with pulmonary TB at eight outpatient clinics in Hanoi, Vietnam. The Uganda study was a random sample of consecutive adults diagnosed with pulmonary TB at Mulago Hospital Complex, Kampala. The Benin sub-study included consecutive participants in the RAFA trial described above.

Ethics oversight

Ethics approval for “Study 36: An Platform for Assessment of TB Treatment Outcomes An Observational Study of Individuals Treated for Pulmonary Tuberculosis” in Vietnam was provided by the following three IRBs: (1) Vietnam Ministry of Health Ethical Committee in National Biological Medical Research (12/QD-BYT), (2) University of California San Francisco Human Research Protection Program Institutional Review Board (H8660-27882-06) and the US Centers for Disease Control and Prevention Institutional Review Board 2 (6560.0)

Ethics approval for the Uganda observational study was provided by the following five review boards: (1) Uganda National Council for Science and Technology (HS 259) (2) Makerere University Faculty of Medicine Research and Ethics Committee (2006-017), (3) Mulago Hospital Institutional Review Board (2006-017), (4) Colorado Multiple Institutions Review Board (10-0290) and (5) University of California San Francisco Human Research Protection Program Institutional Review Board (H8660-27882)

Ethics approval for the RAFA study was provided by the following three review boards: (1) National Ethics Committee for Research in Health, Benin (004 31 March 2011), (2) University of Cape Town Institutional Review Board (153/2011) and (3) London School of Hygiene and Tropical Medicine Ethics Committee (5917). RAFA was prospectively registered with the PanAfrican Clinical Trials Registry (PACTR201105000291300).

Note that full information on the approval of the study protocol must also be provided in the manuscript.
